# Quality Evaluation of the Traditional Chinese Medicine Moutan Cortex Based on UPLC Fingerprinting and Chemometrics Analysis

**DOI:** 10.3390/metabo15040281

**Published:** 2025-04-18

**Authors:** Wentao Fang, Qianqian Song, Han Luo, Rui Wang, Chengwu Fang

**Affiliations:** School of Pharmacy, Anhui University of Chinese Medicine, Hefei 230012, China; fangwentao@ahtcm.edu.cn (W.F.); qianqiansong@ahtcm.edu.cn (Q.S.); luohan@ahtcm.edu.cn (H.L.); 13611528427@gamil.com (R.W.)

**Keywords:** moutan cortex, PCA, PLS-DA, fingerprinting, quality evaluation

## Abstract

Background: This study aims to develop a fingerprint analysis method using ultra-high performance liquid chromatography (UPLC) for Moutan Cortex sourced from different regions. The objective is to establish quality control standards validated through the integration of chemometric methods and component structure theory. Methods: The mobile phase for UPLC consisted of acetonitrile (A) and a 0.1% aqueous formic acid solution (B), with gradient elution set as follows: 0–1 min, 8% A → 15% A; 1–8 min, 15% A → 18% A; 8–10 min, 18% A → 30% A; 10–15 min, 30% A → 35% A; 15–20 min, 35% A → 85% A; 20–21 min, 85% A → 8% A; and 21–26 min, 8% A → 8% A. Chemical markers significantly affecting Moutan Cortex from various regions were screened, and their identification was based on comparison with reference materials and content determination. Results: A total of 15 chemical markers were identified, including gallic acid, oxypaeoniflorin, catechin, methyl gallate, paeonolide, apiopaeonoside, albiflorin, paeoniflorin, benzoic acid, 1,2,3,6-tetra-O-galloyl-D-glucose, 1,2,3,4,6-pentagalloylglucose, mudanpioside C, benzoyloxypaeoniflorin, benzoylpaeoniflorin, and paeonol. These markers align with component structure theory, allowing for an analysis of the structural characteristics of Moutan Cortex from different regions. Conclusions: The findings provide a valuable reference for the future quality evaluation of traditional Chinese medicine preparations, enhancing the understanding of the material basis components in Moutan Cortex from diverse sources.

## 1. Introduction

The medicinal value of peony has been recognized since the pre-Qin period, with the earliest recorded mention of “peony” found in the Shen Nong Ben Cao Jing (Shen Nong’s Classic of Materia Medica). Subsequent ancient texts further documented the medicinal use of peony, with its medicinal part being the Moutan Cortex. The Pharmacopoeia of the People’s Republic of China (2020 Edition)specifies that peony bark refers to the dried root bark of the peony plant (Paeonia suffruticosa Andr.) from the family Ranunculaceae. It is traditionally used for clearing heat and cooling the blood as well as promoting blood circulation and resolving blood stasis [1]. Moutan Cortex is primarily produced in provinces such as Anhui, Shandong, Shanxi, Henan, Hunan, Hubei, Sichuan, Chongqing, Gansu, and Shaanxi. Peony bark is rich in compounds, including phenols and monoterpenes [2,3,4,5,6,7], and the quality of the medicinal material is directly influenced by the concentration of these compounds.

The use of a single or a few quality indicators in the quality assessment of Moutan Cortex is insufficient to comprehensively reflect the overall quality of the medicinal material. Since the effective components of traditional Chinese medicine are often complex multi-component systems, and their therapeutic effects may be related to the combined action of multiple components, relying on a single high-content ingredient to evaluate quality does not accurately represent its therapeutic efficacy. Accordingly, researchers are increasingly using multi-index components to evaluate the overall quality of TCM [8,9,10,11]. For example, Jia Xiaobin’s research group [12,13,14,15] proposed the “component structure theory” advocating the qualitative basis of TCM based on the overall view of it, which offers a novel approach for future research pertaining to TCM quality control. On the other hand, in the 2020 edition of the *Chinese Pharmacopoeia* [16], Moutan Cortex is listed as using only paeonol to control its quality. While this type of evaluation indicator is relatively simple, it is difficult to use in order to evaluate quality overall. Hence, in the present study, we contend that it is necessary to use multiple indicators to control the overall quality of Moutan Cortex.

The “fingerprint” analysis of traditional Chinese medicine provides an effective method for the overall quality assessment and control of Chinese medicine by reflecting the complex relationships among its chemical components. For the purposes of the present study, this approach groups individual elements with high homogeneity into one category, often based on fingerprints, in order that they can be distinguished according to the authenticity, origin, and processing methods of the TCM and the quality of the TCM preparations. Principal component analysis (PCA) [17] is a data evaluation approach based on projection in which an unsupervised model generates a number of new variables by linearly combining the multidimensional variables of the original data matrix with a certain weight. This identification method can simply and intuitively reflect the differences between the samples and is a commonly used fingerprint analysis method in relation to TCM. Finally, partial least squares-discriminant analysis (PLS-DA) [18,19,20] is a statistical analysis method that integrates PCA, canonical correlation analysis, and multiple linear regression analysis. A supervised pattern recognition method based on partial least squares, it has high prediction accuracy and wide applicability and offers certain advantages in the comprehensive analysis of multidimensional information regarding the quality of TCM.

In the present study, ultra-performance liquid chromatography (UPLC) was used to establish the fingerprint of Moutan Cortex sourced from different regions, and then 15 chemical markers were selected, using the chemometrics method, and identified through comparison with reference materials. Guided by component structure theory, this paper explores the unique component structure characteristics of Moutan Cortex from different regions, in order to provide valuable reference for the future quality evaluation of the medicine.

## 2. Materials and Methods

### 2.1. Plant Materials and Reagents

The reference substances, including gallic acid (batch number: 180308, purity ≥ 98%), catechin (batch number: 181210, purity > 98%), paeoniflorin (batch number: 180622, purity ≥ 98%), and PGG (batch number: 181001, purity > 98%), were all purchased from Beijing Century Aoko Biotechnology Co., Ltd. (Beijing, China).; benzoyl-oxy-paeoniflorin (batch number: DST191210-088, purity ≥ 95%), danpianphenol glycoside (batch number: DST200302-049, purity ≥ 98%), peony glycoside C (batch number: DST200226-065, purity ≥ 98%), methyl gallate (batch number: DST200424-077, purity ≥ 98%), and galloyl-paeoniflorin (CAS: 122965-41-7, purity ≥ 98%) were purchased from Chengdu Dest Bio Co., Ltd. (Chengdu, China).; paeonolactone glycoside (batch number: 19122302, purity 98.76%), oxidized paeoniflorin (batch number: 19120604, purity 99.67%), and benzoyl-paeoniflorin (batch number: 20032703, purity > 98%) were purchased from Chengdu Pufide Biotechnology Co., Ltd. (Chengdu, China).; benzoic acid (Guizhou Dida Biotechnology Co., Ltd., batch number: GZDD-0182, purity > 98%), paeonol (Guangzhou Meiyi Biotechnology Co., Ltd., Guangzhou China batch number: 170420, purity > 99%), and new paeonol glycoside (Hefei Bomei Biotechnology Co., Ltd., Hefei China batch number: SX114047, purity > 98%), as well as tetragalloylglucose (Wuhan Tianzhi Biotechnology Co., Ltd., Wuhan China batch number: CFS201901, purity ≥ 98%); acetonitrile (OCEANPAK), methanol (Merck), and formic acid (TCL, purity > 98%) were all of chromatographic purity, while methanol (Wuxi Jiani Chemical Co., Ltd., Wuxi, China) was of analytical grade, and ultrapure water was prepared using a Milli-Q ultrapure water system (Merck Chemical Technology (Shanghai) Co., Ltd. (Shanghai, China).

The Moutan Cortex medicinal material sample was authenticated as genuine by Professor Fang Chengwu from Anhui University of Traditional Chinese Medicine. Specific information can be found in Table 1.

### 2.2. Sample Preparation

Weigh approximately 0.25 g of Moutan Cortex powder (sieved through a No. 4 sieve) precisely, and place it in a stoppered conical flask. Accurately add 25 mL of 70% methanol, stopper the flask tightly, and record the weight. Subject the mixture to ultrasound treatment for 30 min (power: 250 W, frequency: 40 kHz). Allow it to cool, record the weight again, and use 70% methanol to make up the loss in weight. Mix well and centrifuge at 4500 rpm for 10 min. Collect the supernatant and filter it through a 0.22 μm micropore filter membrane to obtain the solution.

### 2.3. UPLC Analysis

The chromatographic column used was an ACQUITY UPLC HSS T3 (100 mm × 2.1 mm, 1.8 µm). The mobile phase consisted of acetonitrile (A) and a 0.1% aqueous formic acid solution (B) with gradient elution as follows: 0–1 min, 8% A → 15% A; 1–8 min, 15% A → 18% A; 8–10 min, 18% A → 30% A; 10–15 min, 30% A → 35% A; 15–20 min, 35% A → 85% A; 20–21 min, 85% A → 8% A; and 21–26 min, 8% A → 8% A. The flow rate was set at 0.3 mL/min, the injection volume was 2 µL, the detection wavelength was 246 nm, and the column temperature was maintained at 30 °C.

### 2.4. Data Analysis

An evaluation of the chromatographic fingerprints was performed using chemometrics methods, incorporating Similarity Evaluation System for Chromatographic Fingerprint of Traditional Chinese Medicine software (version 2004A; National Pharmacopoeia Committee, Beijing, China), CA (using SPSS, version 21.0; IBM, Armonk, NY, USA), PCA, and PLS-DA (using SIMCA, version 14.1; Sartorius Stedim Data Analytics AB, Umeå, Sweden).

## 3. Results and Discussion

### 3.1. Optimum Conditions for UPLC Analysis

Various elution systems, including methanol–water, methanol–0.1% formic acid water, methanol–0.05% formic acid water, acetonitrile–water, acetonitrile–0.1% formic acid water, and acetonitrile–0.05% formic acid water, were compared. The results indicated that using acetonitrile–0.1% formic acid water as the mobile phase provided the best separation and peak shape. Additionally, extraction solvents (methanol, 50% methanol–water, 70% methanol–water), detection wavelengths (200–400 nm), and flow rates (0.2, 0.3, 0.4 mL.min^−1^) were examined. Ultimately, 70% methanol–water ultrasonic extraction, 246 nm as the detection wavelength, and 0.3 mL/min as the flow rate were selected.

### 3.2. Establishing a UPLC Fingerprint of Moutan Cortex

Fourteen batches of Moutan Cortex samples were analyzed using UPLC to establish a characteristic and comprehensive fingerprint, and standardization was performed using the relevant software from the National Pharmacopoeia Committee. A total of 24 common peaks were identified in the UPLC fingerprint, which formed the reference chromatogram. By superimposing the fingerprints of each batch, the sample fingerprints for the 14 batches of Moutan Cortex were ultimately obtained (Figure S1).

### 3.3. Relative Retention Times and Relative Peak Areas

Of the 24 common peaks observed, peak 24 was chosen as the reference peak, due to its high content, high intensity, and moderate retention time values depicted in the Moutan Cortex chromatograms. Relative retention times (RRTs) and relative peak areas (RPAs) with respect to the reference peak were then measured, as the RRT and RPA statistics in the UPLC fingerprints could be used in the quality assessment of Moutan Cortex. The results showed that the relative standard deviation (RSD) of the RRTs of the 24 common peaks in the 14 batches of Moutan Cortex samples was less than 0.94%, whereas the RSD of the RPAs of the common peaks was greater than 15.27%. These results indicate that the RRTs among the common peaks were relatively stable. However, the RSDs of the RPAs were quite different, indicating that there was a certain difference in the content of each component in the Moutan Cortex obtained from different regions.

### 3.4. Similarity Analysis

The similarities, further to calculations comparing the complete chromatographic profiles of the 14 batches of Moutan Cortex and the reference chromatogram, were obtained using the Similarity Evaluation System(version 2004A; National Pharmacopoeia Committee, Beijing, China) for the Chromatographic Fingerprint of Traditional Chinese Medicine software (version 2004A; National Pharmacopoeia Committee, Beijing, China), as recommended by the former State Food and Drug Administration (now the National Medical Products Administration). Correlation coefficients for all 14 samples are shown in Table 2. The similarity values of the 14 batches of samples were found to be between 0.918 and 0.996, indicating that the chemical compositions of the Moutan Cortex samples from different regions were relatively similar.

### 3.5. CA

**Figure 1:**
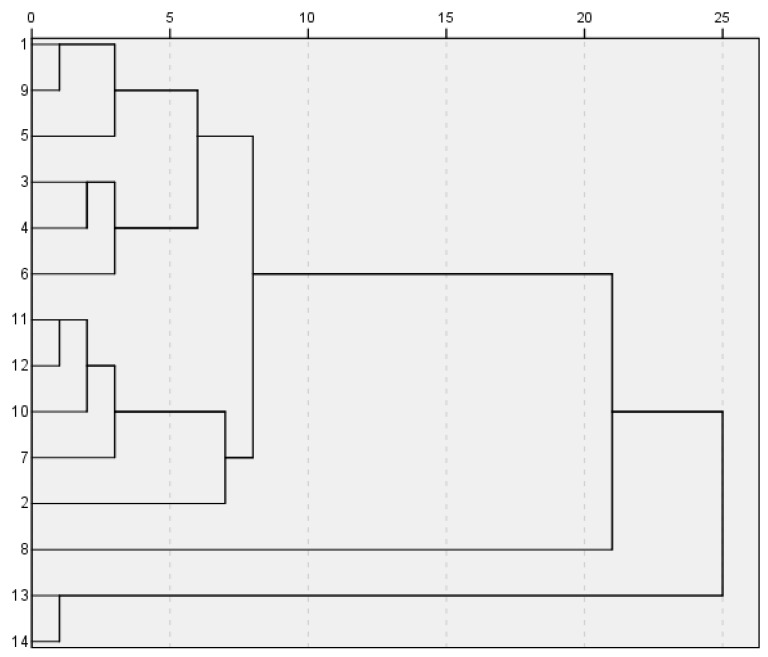
Dendrogram plotting the clustering results for the fingerprints of the 14 batches of Moutan Cortex.

Cluster analysis was carried out using SPSS (version 21.0) software (IBM, Armonk, NY, USA), in order to calculate similarity measures through squared Euclidean distances. The results are presented in a dendrogram (Figure 1), in which the degree of association among samples depends upon distance; the shortest distance shows the highest degree of relationship, and, consequently, those objects are considered to be attributes of the same group. As can be seen in Figure 1, the 14 sample objects grouped into two main clusters among the different regional samples of the Moutan Cortex. The first cluster (Cluster I) contained samples from batches S1 to S12, while the second (Cluster II) consisted of samples from batches S13 and S14. In addition, within Cluster I, batches S1, S3, S4, S5, S6, and S9 showed a smaller distance and were defined as one class; S2, S7, S10, S11, and S12 were another group. These results suggest that the samples of Moutan Cortex from distinct regions have certain differences. (Note that Tongling and Nanling in Anhui province, as the authentic production areas of Moutan Cortex, basically belong to the same category.).

### 3.6. PCA and PLS-DA

**Figure 2:**
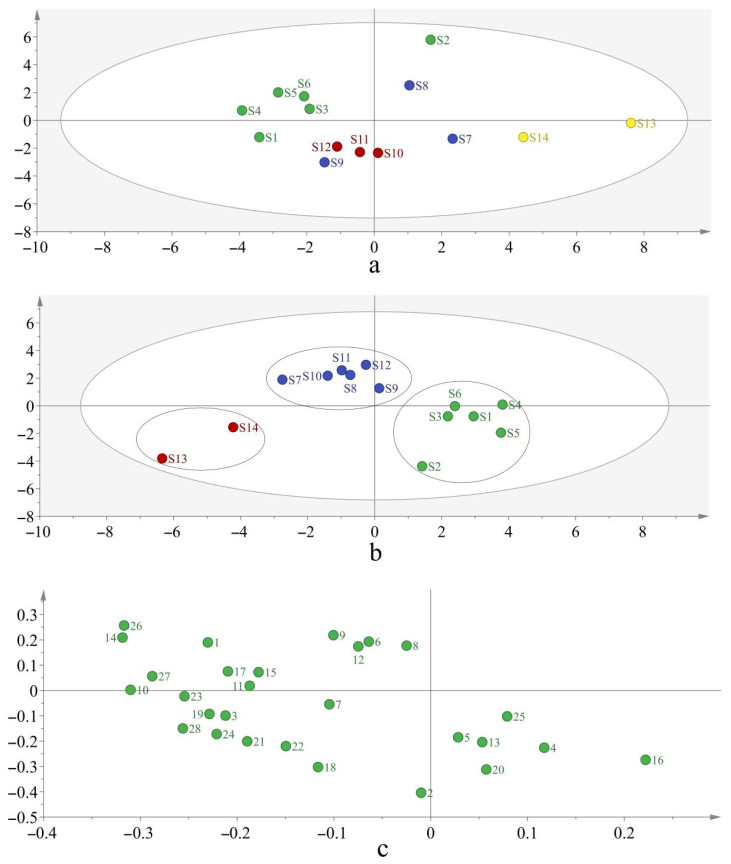
(**a**) Score plot of the PCA for the 14 samples; (**b**) score plot of the PLS-DA for the 14 samples; (**c**) loadings plot of the PLS-DA for the 24 characteristic peaks.

The peak area of the 24 common peaks of the 14 batches of Moutan Cortex samples from different regions was imported as a variable into SIMCA (version 14.1) multivariate statistical analysis software for PCA (Sartorius Stedim Data Analytics AB, Umeå, Sweden). The results show that the four principal components with the largest contribution rate described the 83.60% variability among the samples, indicating that there are significant differences between the Moutan Cortex sample groups from different regions (Figure 2a). In order to ascertain the intergroup differences and component differences in the Moutan Cortex samples from different regions, the PLS-DA model was further established. Four principal components were extracted. The model quality parameter R^2^X was 0.828, R^2^Y was 0.930, and the prediction ability parameter Q^2^ was 0.737, all of which are higher than 0.5, indicating that the established model had strong interpretation and prediction rates.

As shown in the score plot of the PLS-DA (Figure 2b), the Moutan Cortex from Tongling and Nanling (batches S1–S6) are grouped together, and the Moutan Cortex samples from Henan (batches S13 and S14) are grouped together. This result is basically consistent with the CA result.

The Moutan Cortex from Bozhou (S7–S10) and Shandong (S11, S12) are grouped together, probably because the germplasm of Moutan Cortex in Bozhou was introduced from Shandong [21]. According to the results shown in Figure 2c, peaks 1, 2, 3, 4, 5, 7, 8, 10, 14, 15, 16, 19, 20, 22, and 24 may play an important role in distinguishing the differences in the Moutan Cortex samples from different regions.

### 3.7. Identification of Chemical Composition

**Figure 3:**
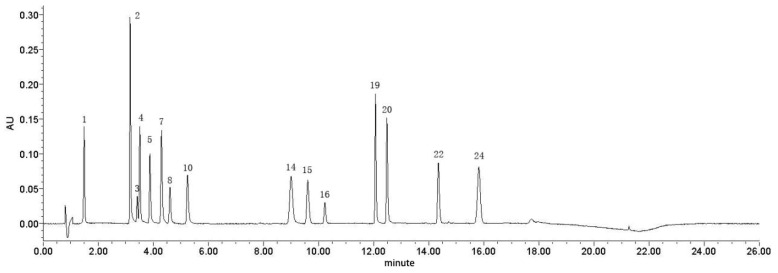
UPLC of mixed-reference substances.

Fifteen characteristic peaks were identified through comparison with reference substances (Figure 3), as follows: gallic acid (peak 1), oxypaeoniflorin (peak 2), catechin (peak 3), methyl gallate (peak 4), paeonolide (peak 5), apiopaeonoside (peak 7), albiflorin (peak 8), paeoniflorin (peak 10), benzoic acid (peak 14), 1,2,3,6-tetra-O-galloyl-D-glucose (peak 15), 1,2,3,4,6-pentagalloylglucose (peak 16), mudanpioside C (peak 19), benzoyloxypaeoniflorin (peak 20), benzoylpaeoniflorin (peak 22), and paeonol (peak 24).

### 3.8. Method Validation

Table 3 presents the results for the validation of the study’s methodology. All calibration curves showed good linearity (*R*^2^ > 0.9970) within the determination range. Satisfactory sensitivity was found in respect of all analytes. The RSDs (%) of the intraday, interday, repeatability, and stability tests of the 15 analytes were 0.52–1.96%, 0.55–2.01%, 0.35–1.98%, and 0.31–1.62%, respectively. The average recovery rate was 96.17% to 111.07%, with a RSD% of 1.68% to 2.41%. These results confirm the reliability of the developed method.

### 3.9. Analysis of Chemical Composition Content

This study screened and characterized the components of the structural features of the Moutan Cortex: namely, those of a terpene nucleoside composition (oxypaeoniflorin, albiflorin, paeoniflorin, mudanpioside C, benzoyloxypaeoniflorin, benzoylpaeoniflorin); phenolic and phenolic glycoside components (paeonolide, apiopaeonoside, paeonol); and those of a tannic acid composition (gallic acid, methyl gallate, benzoic acid, catechin; 1,2,3,6-tetra-O-galloyl-D-glucose, 1,2,3,4,6-pentagalloylglucose). The contents of these 15 characteristic compounds were determined, and our findings are summarized in Table 4.

**Figure 4:**
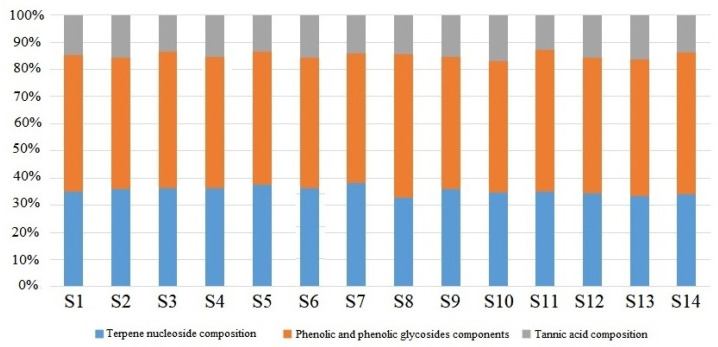
Component percentages of the 14 sample batches of Moutan Cortex.

The mean content of the three components in the 14 batches of Moutan Cortex sample was close to the median, so the mean content of the three components was used to represent the quantity and quantity ratio relationship among terpene nucleoside composition, phenolic and phenolic glycoside components, and tannic acid composition in the Moutan Cortex sample. The quantity and quantity ratio of the components of batches S1–S6 were 24.10 mg/g, 33.44 mg/g, 10.62 mg/g, 1.00:1.39:0.44; for S7–S9, they were 27.55 mg/g, 39.04 mg/g, 11.32 mg/g, 1.00:1.42:0.41; for S10–S12, they were 28.29 mg/g, 40.87 mg/g, 12.40 mg/g, 1.00:1.44:0.44; and for S13–S14, they were 31.60 mg/g, 48.09 mg/g, 14.16 mg/g, 1.00:1.52:0.45. It can be seen that the relationship between the quantity ratio of the components in batches S7–S9 (Bozhou) and S10–S12 (Shandong) is closer to that of batches S1–S6 (Tongling and Nanling), but there is a difference between the quantities of the components. The relationship between the quantity and quantity ratio of the components in batches S13–S14 (Henan) is quite different from that in batches S1–S6 (Tongling and Nanling); the relationship between the quantity and quantity ratio of the components in batches S7–S9 (Bozhou) is similar to that in batches S10–S12 (Shandong). This result is consistent with the results of the PLS-DA analysis, indicating that these 15 characteristic compounds play an important role in the differences between the Moutan Cortex samples from different regions. From the perspective of geographical location, within China, Tongling, Nanling, Bozhou, and Shandong are relatively southeast, while Henan is relatively northwest. Our results suggest that divergent geographical environments correspond to the differences between the Moutan Cortex samples. Relative content percentages for the 14 batches of Moutan Cortex samples are shown in Figure 4.

## 4. Conclusions

UPLC was used to establish the fingerprints of samples of Moutan Cortex from different regions, and similarity analysis, CA, PCA, and PLS-DA were used to analyze the fingerprints of the 14 sample batches. From the 24 common peaks, 15 chemical markers were screened and quantitatively determined. The markers, guided by component structure theory, explored the unique component structure characteristics of Moutan Cortex from different regions and provided a valuable reference for the quality evaluation of this herbal medicine. Given the efficacy and comprehensiveness of the UPLC fingerprinting method, in combination with chemometrics, shown in this study, we further advise that this approach would provide a valuable potential reference for future assessments of the authenticity and quality as well as the development of herbal medicines or other related food and pharmaceutical products.

## Figures and Tables

**Table 1:** Moutan Cortex medicinal material sample information.

No.	Longitude Latitude	Longitude Latitude
S1	117°59′54″	30°52′24″
S2	117°59′18″	30°52′40″
S3	118°01′34″	30°51′20″
S4	118°05′32″	30°49′01″
S5	118°17′48″	30°58′00″
S6	117°59′28″	30°49′17″
S7	115°53′13″	33°58′30″
S8	115°52′43″	33°54′18″
S9	115°57′40″	33°43′14″
S10	117°40′40″	35°20′14″
S11	117°46′21″	35°24′16″
S12	117°45′28″	35°24′32″
S13	112°45′37″	33°28′37″
S14	112°37′42″	33°27′27″

**Table 2:** Similarity evaluation results of 14 batches of Moutan Cortex.

	S1	S2	S3	S4	S5	S6	S7	S8	S9	S10	S11	S12	S13	S14
S1	1.000													
S2	0.958	1.000												
S3	0.975	0.979	1.000											
S4	0.975	0.969	0.987	1.000										
S5	0.987	0.980	0.978	0.969	1.000									
S6	0.944	0.963	0.984	0.983	0.945	1.000								
S7	0.977	0.983	0.988	0.988	0.980	0.972	1.000							
S8	0.839	0.909	0.925	0.906	0.853	0.961	0.904	1.000						
S9	0.988	0.959	0.969	0.976	0.973	0.945	0.985	0.851	1.000					
S10	0.972	0.962	0.977	0.993	0.958	0.975	0.989	0.902	0.985	1.000				
S11	0.966	0.967	0.992	0.988	0.959	0.990	0.984	0.941	0.967	0.987	1.000			
S12	0.959	0.961	0.982	0.991	0.948	0.985	0.985	0.931	0.972	0.996	0.993	1.000		
S13	0.979	0.968	0.970	0.981	0.971	0.956	0.984	0.868	0.990	0.989	0.970	0.978	1.000	
S14	0.985	0.967	0.976	0.982	0.974	0.956	0.988	0.867	0.994	0.991	0.975	0.981	0.996	1.000
R	0.981	0.982	0.993	0.994	0.978	0.984	0.996	0.918	0.985	0.994	0.993	0.992	0.989	0.991

**Table 3:** Linear regression equations, precision, stability, repeatability, and recovery of 15 compounds.

No.	Regression Equation	Linear Range (μg/mL)	R^2^	Precision (RSD%)		Stability	Repeatability	Recovery	
				Intraday	Interday	(RSD%, n = 6)	(RSD%, n = 6)	Mean	RSD%
				(n = 6)	(n = 3)				
peak 1	Y = 6292.3X + 901.11	0.67~67.17	0.9995	1.96	2.01	0.45	1.56	99.00	1.68
Peak 2	Y = 8333.7X + 968.99	0.94~93.73	0.9996	0.55	0.62	0.59	1.98	99.61	2.27
Peak 3	Y = 1474.1X + 2855.2	0.79~78.85	0.9996	1.91	1.98	1.62	1.08	98.22	2.18
Peak 4	Y = 6302.4X + 8720.7	0.69~68.57	0.9997	0.52	0.55	0.63	1.90	96.36	2.19
Peak 5	Y = 3217.4X + 8491.9	1.11~111.40	0.9998	1.18	1.45	0.59	1.48	97.48	1.86
Peak 7	Y = 3016.6X − 1758.8	0.25~250.00	0.9991	0.87	1.12	0.31	0.35	99.17	1.89
Peak 8	Y = 3419.9X + 5805.2	0.67~67.10	0.9998	1.74	1.99	1.21	1.80	101.45	2.07
Peak 10	Y = 2480X + 11,696	1.14~268.00	0.9999	1.07	1.68	1.25	0.90	109.02	2.41
Peak 14	Y = 7161.9X + 10,447	0.77~76.69	0.9998	1.77	1.68	0.65	1.89	109.73	1.90
Peak 15	Y = 3948X + 5354.9	0.23~92.86	0.9997	1.89	1.78	1.16	1.51	98.28	2.19
Peak 16	Y = 3046.2X + 5560.8	0.99~148.50	0.9970	1.45	1.41	0.77	0.69	99.97	2.24
Peak 19	Y = 8668.6X + 7157.3	0.64~64.20	0.9995	1.79	1.81	0.84	1.66	98.82	1.75
Peak 20	Y = 8531.3X + 6545.2	0.63~62.70	0.9999	1.71	1.89	0.95	1.59	97.08	2.24
Peak 22	Y = 4192.7X + 5942.2	0.99~98.93	0.9992	1.75	1.81	0.84	1.81	96.17	1.92
Peak 24	Y = 3438.3X + 24,737	1.48~450.00	1.0000	1.74	1.82	1.00	0.84	111.07	1.90

**Table 4:** The contents (mg/g) of 15 constituents in 14 batches of Moutan Cortex extract (n = 3).

Sample No	Terpene Nucleoside Composition						Phenolic and Phenolic Glycosides Components			Tannic Acid Composition					
	Peak 2	Peak 8	Peak 10	Peak 19	Peak 20	Peak 22	Peak 5	Peak 7	Peak 24	Peak 1	Peak 4	Peak 14	Peak 3	Peak 15	Peak 16
S1	2.66 ± 0.025	0.25 ± 0.001	12.80 ± 0.068	1.16 ± 0.014	0.82 ± 0.002	2.68 ± 0.054	0.53 ± 0.001	1.37 ± 0.014	27.28 ± 0.140	2.06 ± 0.011	1.50 ± 0.011	1.37 ± 0.013	2.50 ± 0.023	0.25 ± 0.008	0.83 ± 0.008
S2	4.87 ± 0.036	0.58 ± 0.002	18.67 ± 0.071	0.85 ± 0.005	1.00 ± 0.014	2.41 ± 0.071	2.32 ± 0.014	6.30 ± 0.062	29.90 ± 0.098	0.96 ± 0.008	2.02 ± 0.019	1.50 ± 0.002	5.00 ± 0.027	0.30 ± 0.009	2.56 ± 0.008
S3	4.08 ± 0.048	0.51 ± 0.002	15.32 ± 0.069	0.88 ± 0.003	0.97 ± 0.011	2.64 ± 0.045	2.39 ± 0.009	6.64 ± 0.061	24.96 ± 0.089	1.59 ± 0.012	1.64 ± 0.017	1.31 ± 0.018	3.49 ± 0.025	0.15 ± 0.009	0.95 ± 0.009
S4	3.23 ± 0.019	0.37 ± 0.003	16.23 ± 0.057	0.79 ± 0.006	0.76 ± 0.007	2.49 ± 0.069	1.45 ± 0.007	4.32 ± 0.052	24.47 ± 0.086	1.49 ± 0.014	1.40 ± 0.021	1.11 ± 0.019	3.74 ± 0.031	0.25 ± 0.009	3.72 ± 0.013
S5	3.50 ± 0.011	0.31 ± 0.004	14.87 ± 0.069	0.90 ± 0.007	0.82 ± 0.006	2.65 ± 0.054	0.75 ± 0.009	1.61 ± 0.009	27.57 ± 0.110	1.50 ± 0.017	1.65 ± 0.022	1.30 ± 0.021	3.23 ± 0.024	0.14 ± 0.008	0.51 ± 0.007
S6	3.46 ± 0.041	0.81 ± 0.002	15.93 ± 0.089	0.83 ± 0.005	0.83 ± 0.009	2.67 ± 0.087	3.70 ± 0.084	10.35 ± 0.076	24.72 ± 0.072	1.38 ± 0.016	1.84 ± 0.016	1.20 ± 0.018	4.37 ± 0.019	0.12 ± 0.007	4.78 ± 0.013
Mean	3.63	0.47	15.64	0.9	0.87	2.59	1.86	5.1	26.48	1.5	1.67	1.3	3.72	0.2	2.23
Median	3.48	0.44	15.62	0.86	0.83	2.65	1.89	5.31	26.12	1.49	1.65	1.3	3.62	0.2	1.76
S7	4.63 ± 0.022	0.53 ± 0.002	19.08 ± 0.025	0.95 ± 0.004	1.14 ± 0.013	4.41 ± 0.054	2.17 ± 0.031	4.46 ± 0.074	32.15 ± 0.170	1.84 ± 0.011	1.77 ± 0.009	1.80 ± 0.017	2.23 ± 0.013	0.03 ± 0.005	3.70 ± 0.013
S8	4.03 ± 0.047	2.66 ± 0.001	18.19 ± 0.089	0.89 ± 0.007	1.16 ± 0.031	4.05 ± 0.079	7.53 ± 0.094	19.52 ± 0.110	22.88 ± 0.190	1.35 ± 0.013	2.02 ± 0.002	1.42 ± 0.016	4.13 ± 0.019	0.05 ± 0.005	4.65 ± 0.019
S9	3.02 ± 0.032	0.32 ± 0.003	12.07 ± 0.065	0.96 ± 0.010	0.85 ± 0.006	3.69 ± 0.081	0.54 ± 0.013	1.36 ± 0.018	26.5 ± 0.160	2.23 ± 0.017	1.44 ± 0.016	1.58 ± 0.019	1.67 ± 0.021	0.05 ± 0.004	2.01 ± 0.024
Mean	3.9	1.17	16.45	0.94	1.05	4.05	3.41	8.45	27.18	1.81	1.74	1.6	2.68	0.04	3.45
Median	4.03	0.53	18.19	0.95	1.14	4.05	2.17	4.46	26.5	1.84	1.77	1.58	2.23	0.05	3.7
S10	3.98 ± 0.021	0.59 ± 0.003	18.70 ± 0.059	0.86 ± 0.007	0.95 ± 0.009	3.96 ± 0.087	2.55 ± 0.014	5.35 ± 0.051	32.75 ± 0.161	2.44 ± 0.021	1.29 ± 0.009	1.36 ± 0.015	2.98 ± 0.023	0.12 ± 0.003	5.91 ± 0.041
S11	4.02 ± 0.054	1.15 ± 0.009	17.76 ± 0.061	0.88 ± 0.008	0.94 ± 0.010	3.43 ± 0.091	3.06 ± 0.021	9.4 ± 0.097	29.4 ± 0.190	2.21 ± 0.019	1.32 ± 0.009	1.20 ± 0.018	3.33 ± 0.027	0.03 ± 0.004	2.37 ± 0.023
S12	4.09 ± 0.046	1.08 ± 0.008	17.48 ± 0.057	0.69 ± 0.009	0.88 ± 0.009	3.4 ± 0.014	3.50 ± 0.011	7.57 ± 0.088	29.04 ± 0.140	2.22 ± 0.018	1.33 ± 0.098	1.30 ± 0.018	2.78 ± 0.026	0.12 ± 0.004	4.89 ± 0.019
Mean	4.03	0.94	17.98	0.81	0.92	3.6	3.03	7.44	30.39	2.29	1.31	1.29	3.03	0.09	4.39
Median	4.02	1.08	17.76	0.86	0.94	3.43	3.06	7.57	29.4	2.22	1.32	1.3	2.98	0.12	4.89
S13	5.29 ± 0.033	0.73 ± 0.004	19.23 ± 0.089	1.32 ± 0.011	1.26 ± 0.011	3.96 ± 0.031	1.84 ± 0.002	3.68 ± 0.032	42.51 ± 0.231	2.12 ± 0.011	1.81 ± 0.010	1.58 ± 0.019	3.45 ± 0.021	0.15 ± 0.004	6.49 ± 0.064
S14	5.33 ± 0.043	0.52 ± 0.004	19.25 ± 0.075	1.10 ± 0.014	1.18 ± 0.012	4.02 ± 0.068	1.82 ± 0.019	3.63 ± 0.030	42.69 ± 0.250	2.17 ± 0.014	1.66 ± 0.011	1.49 ± 0.017	3.31 ± 0.027	0.13 ± 0.034	3.95 ± 0.059
Mean	5.31	0.62	19.24	1.21	1.22	3.99	1.83	3.66	42.6	2.15	1.74	1.53	3.38	0.14	5.22
Median	5.31	0.62	19.24	1.21	1.22	3.99	1.83	3.66	42.6	2.15	1.74	1.53	3.38	0.14	5.22

## Data Availability

The original contributions presented in this study are included in the article/supplementary material. Further inquiries can be directed to the corresponding author(s).

## Supplementary Materials

The following are available online at https://www.mdpi.com/article/10.3390/metabo15040281/s1, Figure S1: Figure S1. (a) The characteristic spectrogram for the chromatographic fingerprint. (b) The chromatographic fingerprints and characteristic peaks of 14 batches of Moutan Cortex.

## Abbreviations

CA, cluster analysis; CAS, Chemical Abstract Services (registry); PCA, principal component analysis; PLS-DA, partial least squares-discriminant analysis; RPA, relative peak area; RRT, relative retention time; RSD, relative standard deviation; UPLC, ultra-performance liquid chromatography.
