# Supplementary material for: Quality Evaluation of the Traditional Chinese Medicine Moutan Cortex Based on UPLC Fingerprinting and Chemometrics Analysis
Source: Metabolites. 2025 Apr 18;15(4):281. doi: 10.3390/metabo15040281 (PMC12029947; doi:10.3390/metabo15040281)
Supplement: Supplementary file 1 [file metabolites-15-00281-s001.zip › metabolites-3541596-supplementary.pdf]

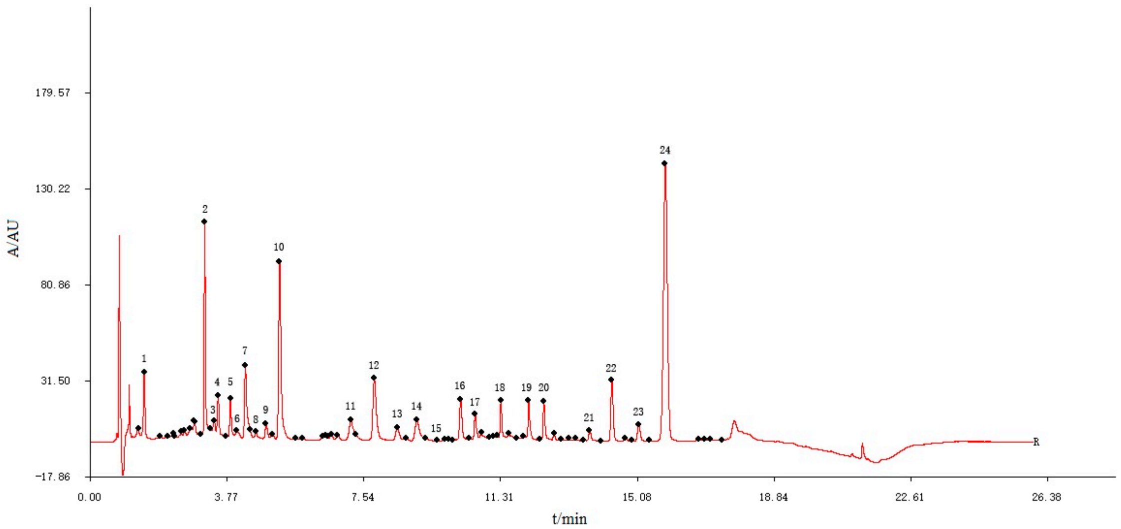

a

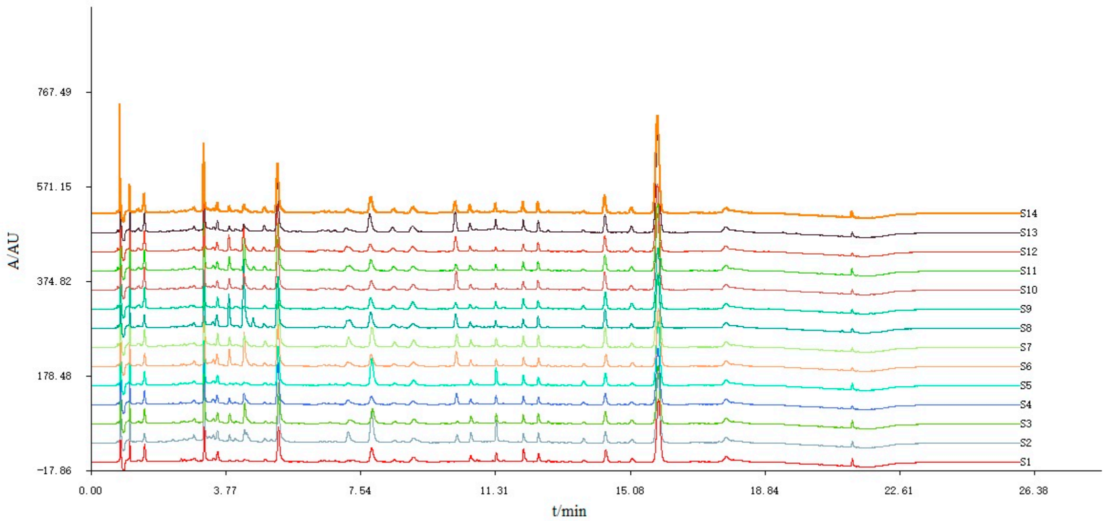

b

Figure S1. (a) The characteristic spectrogram for the chromatographic fingerprint. Components: 1. gallic acid; 2. oxypaeoniflorin; 3. catechin; 4. methyl gallate; 5. paeonolide; 6. unknown; 7. apiopaeonoside; 8. albiflorin; 9. unknown; 10. paeoniflorin; 11. unknown; 12. unknown; 13. unknown; 14. benzoic acid; 15. 1,2,3,6-tetra-O-galloyl-D-glucose; 16. 1,2,3,4,6-pentagalloylglucose; 17. unknown; 18. unknown; 19. mudanpioside C; 20. benzoyloxypaeoniflorin; 21. unknown; 22. benzoylpaeoniflorin; 23. unknown; 24. paeonol. (b) The chromatographic fingerprints and characteristic peaks of 14 batches of Moutan Cortex (samples 1 to 14 labeled “S1,” “S2,” etc.).
